# High mitochondrial calcium levels precede neuronal death *in vivo* in Alzheimer's disease

**DOI:** 10.15698/cst2020.07.226

**Published:** 2020-06-18

**Authors:** Maria Calvo-Rodriguez, Brian J. Bacskai

**Affiliations:** 1 Alzheimer Research Unit, Department of Neurology, Massachusetts General Hospital and Harvard Medical School, 114, 16th St, Charlestown, MA 02129, USA.

**Keywords:** calcium homeostasis, mitochondria, Alzheimer's disease, amyloid, mitochondrial calcium uniporter

## Abstract

Alzheimer's disease (AD), the most common cause of dementia, affects millions of people worldwide. Suggested mechanisms of neurotoxicity in AD include impaired calcium (Ca^2+^) homeostasis and mitochondrial dysfunction, both contributing to neuronal damage. Little was known about the exact mitochondrial Ca^2+^ homeostasis in the living brain, particularly in AD. Only now, with the development of intravital imaging techniques and transgenic mouse models of the disease, we are able to directly observe Ca^2+^ levels in specific regions or particular subcellular compartments of cells, such as mitochondria. Using multiphoton microscopy, a Ca^2+^ reporter targeted to mitochondria and a mouse model of cerebral β amyloidosis (APP/PS1), our recent study (Nat Comms 2020, 11:2146) found elevated mitochondrial Ca^2+^ concentration in the transgenic mouse after plaque deposition, and after topical application of natural soluble amyloid beta (Aβ) oligomers to the healthy mouse brain at concentrations similar to those found in the human brain. Elevated Ca^2+^ in mitochondria preceded neuronal death and could be targeted for neuroprotective therapies in AD. Here, we describe our main findings and pose new questions for future studies aimed at better understanding mitochondrial Ca^2+^ dyshomeostasis in AD.

## MITOCHONDRIAL CALCIUM OVERLOAD AFTER PLAQUE DEPOSITION

**Figure 1:**
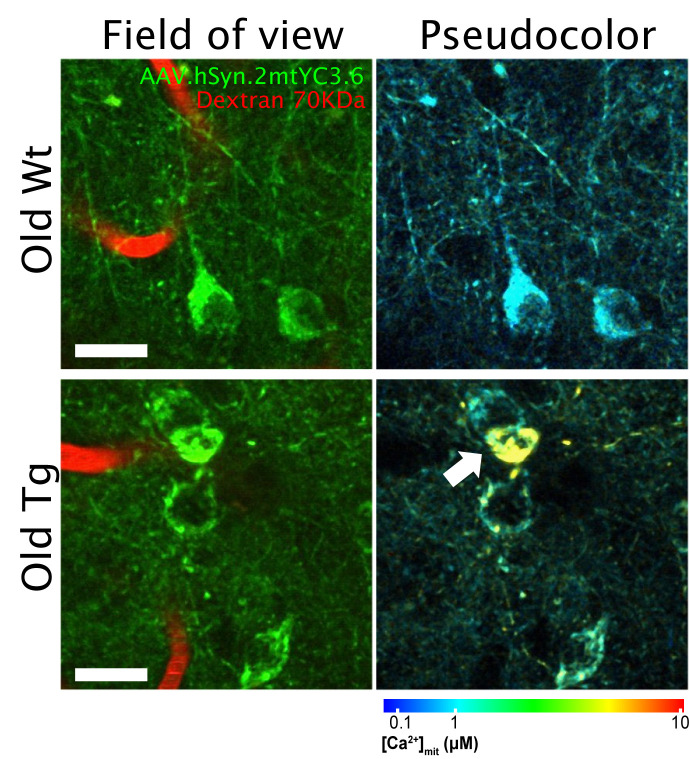
FIGURE 1: *In vivo* multiphoton microscopy images of mitochondrial Ca^2+^ in neurons in the Wt and APP/PS1 mice. Mice were injected with AAV.hSyn.2mtYC3.6 and a cranial window was implanted. Mitochondrial Ca^2+^ was later evaluated with multiphoton microscopy. Pictures are representative of old wild-type (Wt, top) and transgenic mice (APP/PS1 Tg, bottom). The field of view shows the AAV transduction in neuronal mitochondria (green) and the blood vessels labeled with fluorescent Dextran (red). Pseudocolor images represent the color coded mitochondrial Ca^2+^ concentrations for the corresponding field of view images. Arrow points to a neuron with mitochondrial Ca^2+^ overload. Scale bar represents 20 μm.

Ca^2+^ is essential for the normal function of mitochondrial activity, and therefore Ca^2+^ levels are tightly regulated in mitochondria. However, excessive levels of Ca^2+^ in mitochondria, i.e. mitochondrial Ca^2+^ overload, can lead to an increase of reactive oxidative stress (ROS) species, decrease of ATP production and eventually to cell death via apoptosis. Cytosolic Ca^2+^ homeostasis is impaired in AD, as evidenced by hyperactive neurons close to amyloid plaques or high cytosolic Ca^2+^ in transgenic mice with plaque deposition. But, is mitochondrial Ca^2+^ homeostasis also impaired *in vivo* in AD? Are Ca^2+^ levels increased or diminished in the neurons? *In vitro* studies suggested that mitochondrial Ca^2+^ homeostasis was altered in the presence of Aβ oligomers (Aβo), but these questions remained unresolved *in vivo*. To address this issue, we used a transgenic mouse model of cerebral amyloidosis, APP/PS1 – a model that develops amyloid plaques comparable to those from human patients starting at around four months of age – and intravital imaging with multiphoton microscopy. To measure Ca^2+^ concentration in mitochondria, we used a ratiometric Ca^2+^ reporter (Yellow Cameleon, YC) targeted to neuronal mitochondria (hSyn.2mt.YC3.6), that we injected as adenovirus (AAV) in the somatosensory cortex of the mice. This ratiometric reporter, based on Förster resonance energy transfer (FRET), allows for quantitative measurement of absolute Ca^2+^ concentration and direct comparison between cells and compartments. We observed mitochondrial Ca^2+^ overload in the neurons of old APP/PS1 transgenic mice, once plaque deposition had started, unlike the young transgenic mice (pre-pathology). Transgenic mice showed twice as many mitochondria with high Ca^2+^ compared to the wild-type (Wt) mice. This difference was found in both somas and neurites. Other studies have previously reported no changes in cytosolic Ca^2+^ or neuronal function before plaque deposition, supporting the notion that mitochondrial Ca^2+^ overload in neurons follows, rather than precedes, amyloid plaque deposition. **Figure 1** shows an example of *in vivo* multiphoton microscopy images of mitochondria in neurons in APP/PS1 mouse and in Wt mouse after transduction of the AAV. We did not find any correlation between mitochondrial Ca^2+^ overload and the distance to plaques, suggesting that diffusible soluble Aβ species (Aβ oligomers, Aβo), rather than individual plaques, are responsible for the mitochondrial Ca^2+^ increase in the neurons of the transgenic mouse.

## SOLUBLE Aβ OLIGOMERS INCREASE MITOCHONDRIAL CALCIUM IN NEURONS VIA THE MCU

To confirm that soluble Aβ species drive the mitochondrial Ca^2+^ increase in neurons, we topically applied naturally secreted Aβo (obtained from primary cortical neurons isolated from transgenic embryos, referred to as transgenic conditioned media, TgCM) at concentrations similar to those found in the human AD brain directly onto the surface of the living brain of a Wt naïve mouse (C57BL/6J) expressing the adenovirus hSyn.2mtYC3.6. Application of TgCM transiently increased mitochondrial Ca^2+^ concentration in the neurons, whereas Wt conditioned media (obtained from Wt littermates) or Aβ-immunodepleted TgCM did not. Neurites were more susceptible to this increase than somas. These results confirmed our previous observations in the APP/PS1 transgenic mouse, and implicated soluble Aβo in the mitochondrial Ca^2+^ dyshomeostasis *in vivo*. After returning to normal mitochondrial Ca^2+^ levels, we observed that mitochondrial shape and size were altered – mitochondria were smaller and rounded –, suggesting that the toxic effects of Aβo *in vivo* were not only restricted to mitochondrial Ca^2+^, but also to mitochondrial morphology. Additionally, this suggests that the change in size and shape is a secondary event after a mitochondrial Ca^2+^ increase. It is believed that mitochondrial fragmentation precedes neuronal cell death, which establishes a connection with mitochondrial Ca^2+^. Interestingly, the APP/PS1 transgenic mouse also exhibits fragmented and swollen mitochondria once the pathology is present, and the brains from AD patients show impaired balance of mitochondrial fission and fusion proteins.

How does Aβ induce mitochondrial Ca^2+^ uptake? And is it possible to inhibit it in order to prevent or decrease the mitochondrial Ca^2+^ overload? The mitochondrial Ca^2+^ uniporter (MCU) complex is the main pathway for mitochondrial Ca^2+^ influx. However, the mechanism by which Aβ induces a Ca^2+^ increase in neuronal mitochondria *in vivo* was not previously known. Various hypotheses proposed that Aβ could create pores in membranes, form a Ca^2+^-permeable pore itself or act on native channels. In this study, we demonstrated that blocking the MCU complex *in vivo* with Ru360 – a specific blocker of the channel – prevented the mitochondrial Ca^2+^ uptake elicited by TgCM. To confirm that Ru360 application did not render neurons insensitive to Aβ or blocked the Ca^2+^ influx channels of the plasma membrane, we measured the cytosolic Ca^2+^ influx induced by Aβo with the same Ca^2+^ reporter (YC) targeted to the cytosol of neurons (CBA.YC3.6). We observed that Ru360 did not interfere with the rise in the cytosolic Ca^2+^ induced by Aβ. These results suggest that MCU is required for the increase in mitochondrial Ca^2+^ induced by Aβ *in vivo*, and points to MCU as a potential target candidate for AD.

## HIGH MITOCHONDRIAL CALCIUM PRECEDES NEURONAL DEATH *IN VIVO*

What are the implications for increased neuronal mitochondrial Ca^2+^ in the *in vivo* brain? We observed that in the APP/PS1 transgenic mouse, the small fraction of neurons with elevated mitochondrial Ca^2+^ in the soma died within 24 hours. Their nuclei became condensed (a sign of apoptosis) and the AAV fluorescence in the soma was lost. This effect did not happen in cells with normal mitochondrial Ca^2+^ levels. Rapid neuronal death was previously observed in the APP/PS1 model following increased oxidative stress in the cytosol. Our *in vitro* data showed that, in addition, soluble Aβ triggered opening of the mitochondrial permeability transition pore (mPTP), a decrease of the mitochondrial membrane potential and activation of caspases, events that were secondary to the increase in mitochondrial Ca^2+^. All these findings together link high mitochondrial Ca^2+^, oxidative stress and neuronal cell death via apoptosis *in vivo* in AD.

## MITOCHONDRIAL CALCIUM DYSREGULATION IN THE HUMAN ALZHEIMER'S BRAIN

Lastly, is mitochondrial Ca^2+^ homeostasis impaired in the human Alzheimer's brain? Because direct analysis of mitochondrial Ca^2+^ levels in the human brain is not feasible, could we evaluate the proteins or channels involved in mitochondrial Ca^2+^ transport in the human brain? And if we can, to what extend are they affected? Using data publicly available at the AMP-AD (Accelerating Medicines Partnership-Alzheimer's disease) knowledge portal, we evaluated the differential expression of genes encoding proteins involved in the mitochondrial Ca^2+^ transport. Ca^2+^ influx into mitochondria is mainly led by the MCU complex, which includes the MCU pore, the regulatory subunits MICU1 and MICu2, the essential regulator EMRE, and other proteins such as MCUb and MICU3. Ca^2+^ efflux from mitochondria is primarily controlled by the Na^+^/Ca^2+^ exchanger (NCLX). We compared the expression of the genes encoding these proteins between AD subjects and control individuals using microarray and RNA-seq datasets from the MSBB (Mount Sinai Brain Bank) and the ROSMAP (Religious Orders Study and Memory and Aging Project) cohorts. We observed that the MCU and all subunits of the complex – Ca^2+^ influx – were downregulated in AD compared with control subjects, whereas NCLX – Ca^2+^ efflux – was the only one upregulated. These results suggest a dysregulation in the human genes encoding for mitochondrial Ca^2+^ transport, and imply a counteracting effect to avoid mitochondrial Ca^2+^ overload in the neurons in the AD human brain.

**Figure 2:**
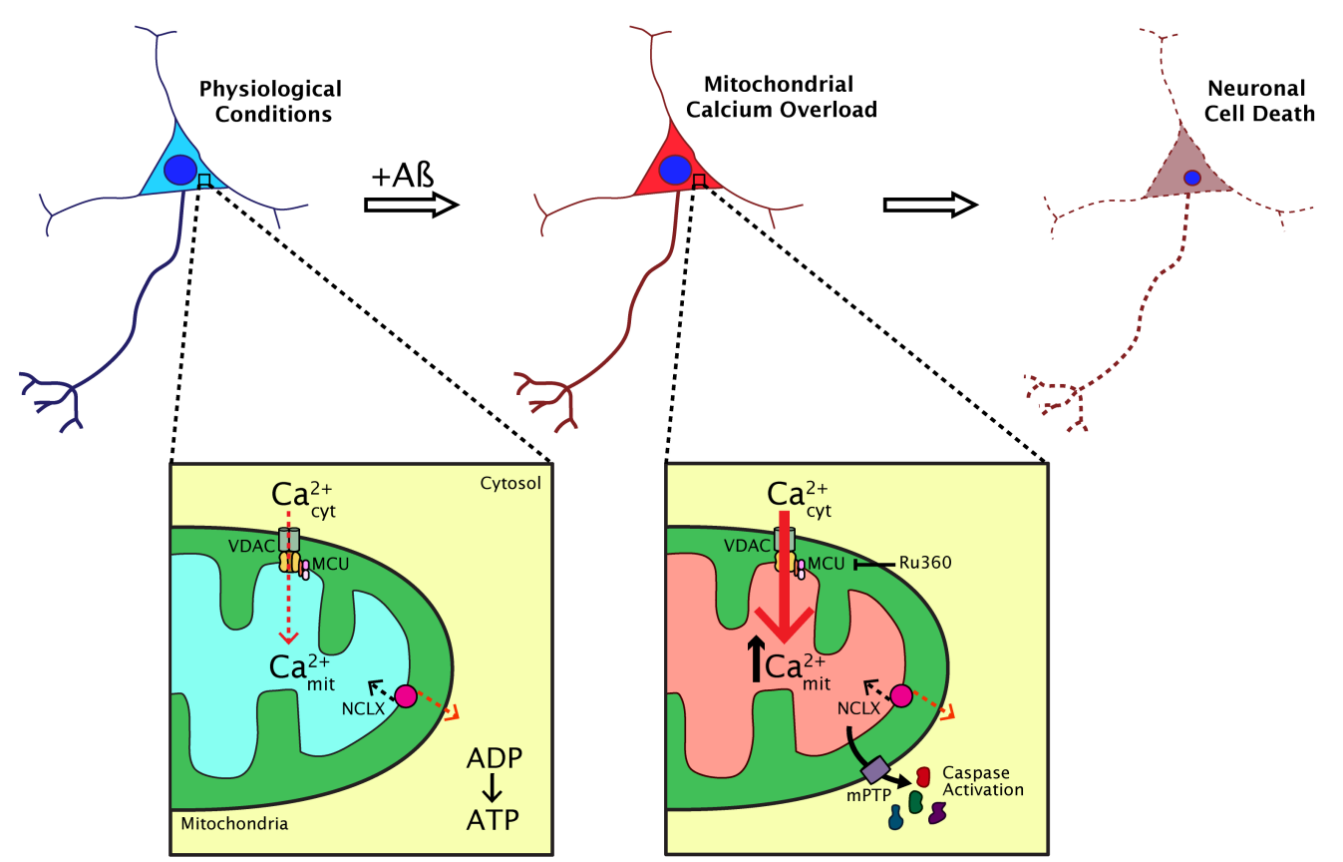
FIGURE 2: Major effects of Aβ aggregates on mitochondrial Ca^2+^ homeostasis in neurons. In physiological conditions, mitochondria take up Ca^2+^ from the cytosol via the MCU to produce ATP among other functions. In AD, Aβ aggregates (particularly soluble Aβ) increase mitochondrial Ca^2+^ via the MCU *in vivo*. This leads to mitochondrial Ca^2+^ overload and activates mPTP and caspases, which can trigger neuronal cell death via apoptosis. Blocking the MCU with Ru360 inhibits mitochondrial Ca^2+^ uptake, pointing to the MCU as a potential target for therapeutics aimed at preventing or reversing the progression of AD.

This study helped clarify the understanding of the dynamics of mitochondrial Ca^2+^ in AD. It suggests that Aβ aggregates (particularly soluble Aβ) increase mitochondrial Ca^2+^ in AD via the MCU *in vivo*, an effect that is linked to neuronal cell death via apoptosis (**Figure 2**). We demonstrated that the extent of mitochondrial Ca^2+^ alterations are spatially restricted in the living brain and highlight the need for imaging studies that can probe these anatomically specific effects. However, this work highlights other questions that remain unresolved. Does mitochondrial Ca^2+^ elevation occur concomitantly with plaque formation? Is it possible to observe mitochondrial morphological abnormalities and altered dynamics (fission, fusion and transport) induced by Aβ *in vivo*? Does tau have any effect on mitochondria, both in terms of Ca^2+^ abnormalities and motility? Additionally, our studies point to MCU as a possible therapeutic target for AD and other neurodegenerative diseases in which mitochondrial Ca^2+^ is impaired, however, further efforts will be needed to unveil the clinical relevance of our findings.

